# Evidence-Based Toxicology—Hypothesis Testing in Randomized Clinical Trials: Part III – Non-Inferiority

**DOI:** 10.1007/s13181-025-01111-7

**Published:** 2025-12-29

**Authors:** Joshua Trebach, Ali Graebner, Mark K. Su

**Affiliations:** 1 Department of Emergency Medicine, Division of Medical Toxicology, University of Iowa Hospitals and Clinics, Iowa City, IA USA; 2 Iowa Poison Control Center, Sioux City, IA USA; 3 New York-Presbyterian Brooklyn Methodist Hospital, Brooklyn, NY USA; 4 Division of Medical Toxicology, Ronald O. Perelman Department of Emergency Medicine, NYU Grossman School of Medicine, New York, NY USA; 5 Department of Health and Mental Hygiene, New York City Poison Control Center, New York, NY USA

**Keywords:** Non-inferiority trial, Clinical trial, Randomized clinical trial

“Do not let the perfect be the enemy of the good.” — *Voltaire*

Non-inferiority trials are studies that are designed to determine if a new treatment is an acceptable alternative treatment by demonstrating that the new treatment is not worse than (i.e., not inferior to or not *unacceptably worse*) the typical or gold-standard treatment. These types of trials are ideal to perform for a variety of reasons. For example, if an investigator wanted to study a shorter course of antibiotics to treat pneumonia, it would be unethical to do this trial with a placebo of no antibiotics—however, the investigator could compare a short course of antibiotics to standard course treatment using a non-inferiority design [1]. It could also be reasonable to perform a non-inferiority trial when a new treatment might not be expected to be superior to the gold standard treatment but the advantage could be that the new treatment is cheaper or has a better side effect profile [2]. In some cases, investigators begin testing a new treatment by attempting to demonstrate non-inferiority before trying to do a superiority trial.

In the world of Medical Toxicology, a non-inferiority trial could theoretically be performed for the treatment of acetaminophen toxicity. For example, if a researcher wanted to perform a non-inferiority trial between a hypothetical new drug (WAC) compared to N-acetylcysteine (NAC) for the treatment of acetaminophen toxicity, the study could then be set up as follows:

- NAC: N-Acetylcysteine (standard, existing drug)
- WAC: Hypothetical new drug
- Null Hypothesis (H_0_) = WAC is not non-inferior to NAC in treating acetaminophen toxicity
- Alternative Hypothesis (H_a_) = WAC is non-inferior to NAC in treating acetaminophen toxicity

In a non-inferiority trial, the alternative hypothesis is the new treatment is *non-inferior* to the standard treatment.

A common misconception regarding non-inferiority trials is that when the null hypothesis is not rejected, the new drug (i.e., WAC) is equal to or better than NAC. However, a non-inferiority trial only determines whether or not the new drug is non-inferior to the standard drug, not if the new drug is equivalent or superior to the standard drug.

Before conducting the non-inferiority trial of WAC compared to NAC, we must define our non-inferiority margin (δ) and state that WAC is non-inferior to NAC if it performs above the non-inferiority margin. When our study is complete, we can compare the mean difference in performance between our new drug WAC and our standard drug NAC (represented on the figure below as a blue circle) and the corresponding confidence intervals. Keep in mind that, in general, sample sizes for non-inferiority trials may be larger than superiority trials and are heavily dependent on effect size chosen by the investigators. It is important to choose accurate, measurable, and feasible primary outcomes when attempting to determine non-inferiority.

It is worth noting here that equivalence and non-inferiority trials are similar however, these two study designs vary in multiple ways. The null hypothesis in an equivalence trial is that the new treatment is not equivalent to the standard treatment, or that the difference between the new and standard treatment is outside the equivalence margin. The alternative hypothesis in an equivalence trial is that the new and standard treatment exists within the equivalence margin. In contrast, the null hypothesis in a non-inferiority trial is that the new treatment is not non-inferior to the standard treatment, or the new treatment is worse than the standard treatment by more than the non-inferiority margin. The alternative hypothesis in a non-inferiority trial is that the new treatment is not worse than the standard treatment by more than the inferiority margin. These are tricky but critical differences; ultimately, the terms equivalence and non-inferiority should not be used interchangeably.

**Fig. 1:**
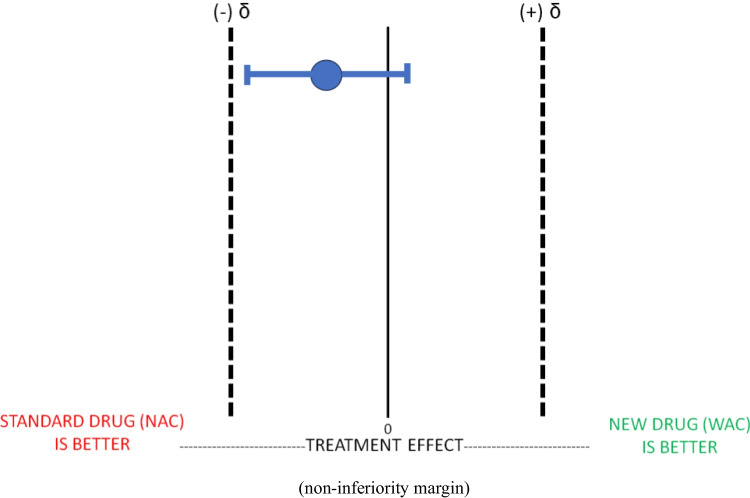
Non-Inferiority Trial: WAC Not Inferior to NAC. Legend: In a Non-Inferiority Trial, for the new experimental drug WAC to be considered non-inferior to the gold standard treatment NAC, the mean treatment effect and it’s CI (typically 95%) must be between the a priori estimated effect size difference (δ), or non-inferiority margin. In this example, WAC demonstrates non-inferiority to NAC because it meets this definition

This figure above (Fig. 1) represents that WAC is non inferior to NAC as our mean value and our confidence intervals are above our non-inferiority margin.

**Fig. 2:**
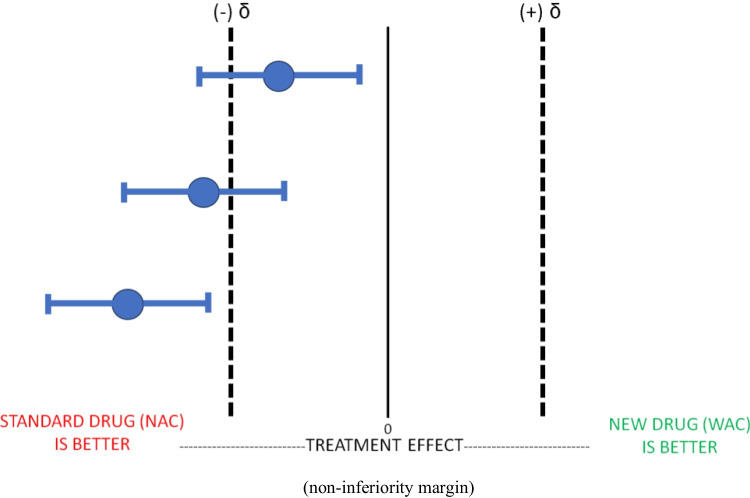
Non-Inferiority Trial: WAC Not Non-Inferior to NAC. Legend: This figure illustrates the results of three separate non-inferiority trials comparing the experimental drug WAC to the gold standard therapy NAC. In Non-Inferiority Trials, the new experimental drug WAC cannot be considered non-inferior to the standard treatment NAC in any of these three trials because the mean treatment effects and their CIs are either overlapping with or, are below the a priori estimated effect size difference/zone of indifference (δ) or less than the estimated effect size difference/zone of indifference (δ). All three trials would therefore be considered “not non inferior”

The figure above represents multiple iterations where WAC is not non-inferior to NAC as our mean value and/or their confidence intervals are below the non-inferiority margin. As noted previously, lack of non-inferiority does not mean that WAC is equivalent or superior to NAC (Fig. 2).

Non-inferiority trials can be used to determine whether a new drug/treatment has advantages over the current gold standard treatment with respect to safety, convenience or cost [3]. The non-inferiority study design has potential inherent challenges that must be considered. Determining a non-inferiority margin could prove to be just as challenging as deciding an equilvance margin. Proper justification of the non-inferiority margin is critical as poorly defined margins can undermine the validity and relevance of the trial. If the non-inferiority margin is too wide, there is a risk of accepting a treatment as being non-inferior when it actually may not be. Conversely, if the non-inferiority margin is too narrow, the trial may require very large and potentially unrealistic sample sizes. Non-inferiorty trials also rely heavily on the fact that the standard treatment is effective. For example, if you find that a new drug is non-inferior to a drug that is the standard treatment but doesn’t work well at all, then by demonstrating that the new drug is non-inferior only results in finding two drugs that both do not work well. In the end, non-inferiority trials are becoming increasingly common and is is imperative for readers to understand this particular study design (Table 1).

**Table 1:** Comparison of the three common randomized controlled trial designs: superiority vs equivalence vs non-inferiority

	Superiority	Equivalence	Non-inferiority
**Objective**	To see if a new treatment is better than what currently exists (the “gold standard”)	To see if a new treatment shows similar equivalence to what currently exists (the “gold standard”)	To see if a new treatment is non inferior to what currently exists (the “gold standard”)
**Null Hypothesis**	New treatment is not superior to current treatment	New treatment is not equivalent to the current treatment	New treatment is not non-inferior to current treatment
**Alternative Hypothesis**	New treatment is superior to current treatment	New treatment is equivalent to the current treatment	New treatment is non-inferior to current treatment
**Sample size needed**	Large	Larger	Largest
**Margin**	Effect size (must be determined prior to conducting the trial)	Equivalence margin (must be determined prior to conducting the trial)	Non-inferiority margin (must be determined prior to conducting the trial)
